# Video-assisted mediastinoscopy (VAM) for surgical resection of ectopic parathyroid adenoma

**DOI:** 10.1186/1749-8090-2-41

**Published:** 2007-10-15

**Authors:** Peter Tcherveniakov, Ashvini Menon, Richard Milton, Kostas Papagiannopoulos, Mark Lansdown, James AC Thorpe

**Affiliations:** 1 Department of Thoracic Surgery, St. James's University Hospital, Leeds LS9 7TF, UK; 2 Department of Breast and Endocrine Surgery, St. James's University Hospital, Leeds LS9 7TF, UK

## Abstract

**Background:**

Ectopic mediastinal parathyroid adenomas or hyperplasia account for up to 25% of primary hyperparathyroidism (HPT). Two percent of them are not accessible by standard cervical surgical approaches. Surgical resection has traditionally been performed via median sternotomy or thoracotomy and more recently, via video assisted thoracoscopic surgery (VATS). We present our experience with the novel use of Video-Assisted Mediastinoscopy (VAM) for resection of ectopic mediastinal parathyroid glands.

**Case presentation:**

4 patients underwent VAM for removal of an ectopic intramediastinal parathyroid gland. All of them had at least one previous unsuccessful neck exploration.

In all cases histology confirmed complete resection of ectopic parathyroid glands (3 parathyroid adenomas and one parathyroid hyperplasia). Two of the patients required a partial sternal split to facilitate exploration.

**Conclusion:**

The cervical approach for resection of ectopic parathyroid adenomas is frequently unsuccessful. Previously, the standard surgical approach in such cases was sternotomy and exploration of the mediastinum. Recently, a number of less invasive modalities have been introduced.

We found that VAM has several advantages. It has a short theatre time does not require a complex anaesthetic and is performed with the patient in classic supine position utilising often a previous cervical scar with good cosmetic results. It offers a short hospital stay; it is cost effective with minimal use of fancy and pricy consumables with a comfortable incision and no violation of the pleural space.

Additionally the use of digital Video imaging has increased the sensitivity of the mediastinoscopy and has added safety and confidence in performing a detailed mediastinal exploration with an additional great training value as well.

## Background

Primary hyperparathyroidism (HPT) is characterized by excessive secretion of parathyroid hormone (PTH), and is a leading cause of hypercalcaemia and hypophosphataemia. Eighty-five percent of primary HPT is caused by parathyroid adenomas and 15% by parathyroid hyperplasia. It has been estimated that 11% to 25% of all patients with primary HPT exhibit ectopic mediastinal hyper functioning parathyroid tissue [1,2]. The treatment consists of surgical excision via cervical incision.

However in 2% of surgical candidates the ectopic parathyroid tissue is not accessible via this approach as the gland is deeply embedded in the superior aspect of the anterior or posterior mediastinum and in close contact with the thymus [3]. These cases have been traditionally treated with median sternotomy, thoracotomy and, less invasively, via video assisted thoracoscopic surgery (VATS) [4]. We would like to present a novel approach with the use of VAM for surgical removal of ectopic parathyroid glands.

## Case presentation

**Figure 1:**
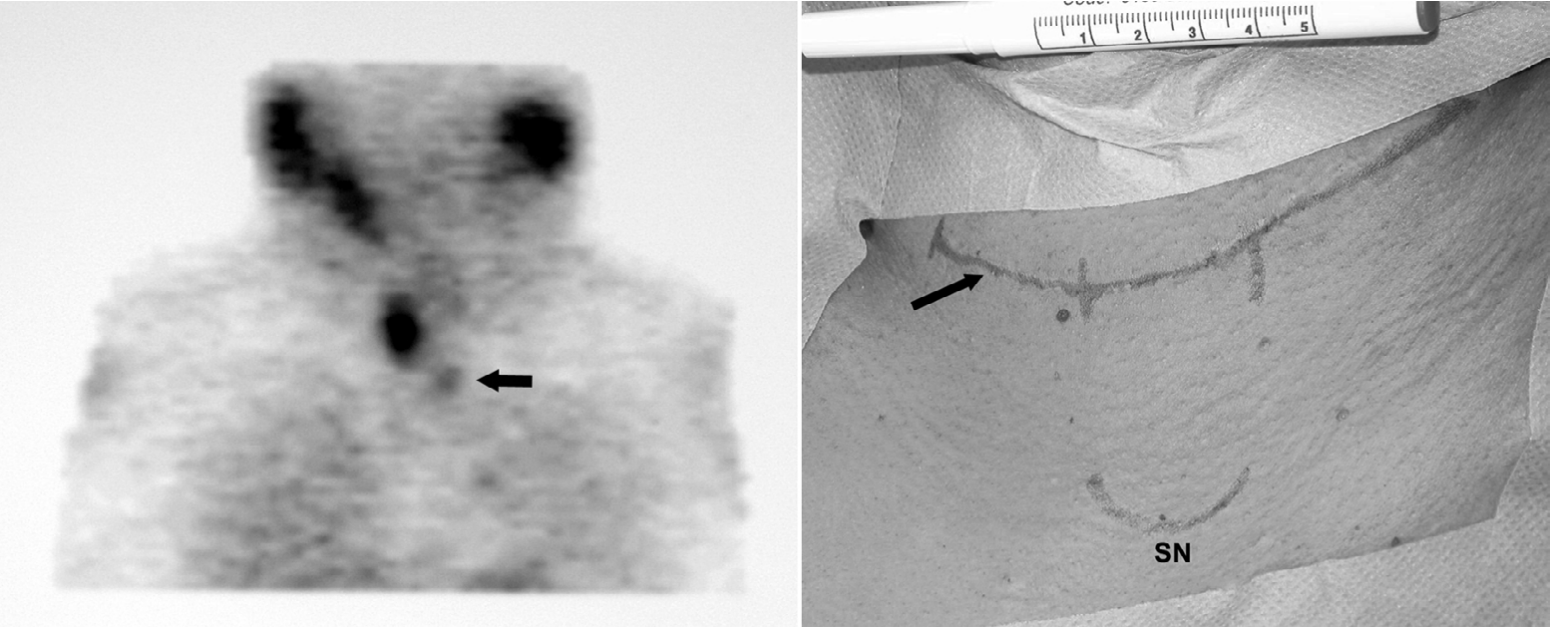
Preoperative imaging with ^99 m ^Tc sestamibi scan (left). Utilization of previous scar (arrow). Extent of incision used for VAM (vertical lines) and its relation to the sternal notch (SN) (right).

**Figure 2:**
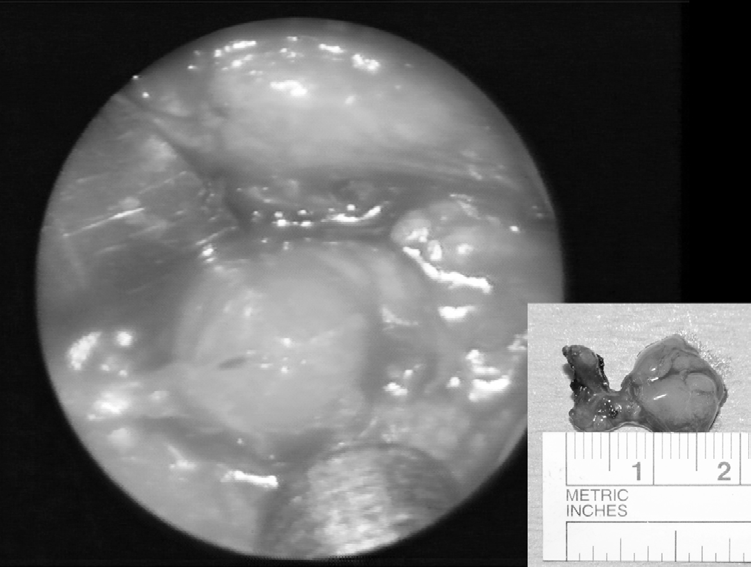
Intraoperative appearance of parathyroid adenoma and resected specimen (inset).

4 patients (1 male, 3 female), with a mean age of 43 years underwent VAM for removal of an ectopic intramediastinal parathyroid gland. All of them had at least one previous unsuccessful neck exploration. Preoperative biochemistry showed mean serum calcium levels of 2.81 mmol/l. All of the patients underwent computed tomography and technitium-99 sestamibi scans for precise preoperative localization of the lesion (Fig. 1). The VAM was performed in the following steps:

• Part of the previous neck incision was utilised (Fig. 1) and a plane immediately behind the manubrium was developed.

• The anterior mediastinal structures were visualised using a Karl-Storz^® ^video-mediastinoscope.

• The ectopic parathyroid was identified and removed using blunt dissection and suction diathermy (Fig. 2).

• Serum ionized calcium was measured to confirm successful localization and excision, and was monitored daily post operatively.

In all cases histology confirmed complete resection of ectopic parathyroid glands (3 parathyroid adenomas and one parathyroid hyperplasia). Two of the patients required a partial sternal split to facilitate exploration.

The mean operative time was 64 minutes. There were no postoperative complications and all patients had an uneventful recovery. Postoperative pain was mild in all cases and controlled by oral analgesia. The mean reduction in serum calcium levels on the first postoperative day was 0.45 mmol/L. All patients reported a perioral tingling sensation on the first day after surgery and were discharged on calcium replacement medication. The mean hospital stay was 2.5 days.

## Conclusion

The most frequent cause of hypercalcaemia is primary hyperparathyroidism. The standard treatment is surgical excision of the responsible gland via a cervical approach without preoperative imaging. However, in about 2% of the cases, the ectopic intramediastinal parathyroid tissue is not accessible with this incision, rendering the procedure inadequate.

Precise preoperative localisation of the ectopic parathyroid adenoma is vital for successful surgical resection. A combination of computed tomographic and ^99 m ^Tc sestamibi scans is the most commonly used and offers the best chance of precise and accurate mapping [1]. The majority of the ectopic parathyroid adenomas are found in the anterior mediastinum, adjacent to or within the residual thymic tissue. Our experience confirms this, with one being intrathymic and the remaining three parathymic in location.

The cervical approach is frequently unsuccessful (all of our cases had previous neck exploration). Previously, the standard surgical approach in such cases was sternotomy and exploration of the mediastinum. Recently, a number of less invasive modalities have been introduced, namely angiographic ablation, VATS [4] and VAM [5]. Angiographic ablation is by far the least invasive, but is unsuccessful in 40% of the cases. Resection of ectopic parathyroid tissue with VATS has been shown to be safe and with good results, but requires selective single lung ventilation and often chest drain insertion with multiple port incisions adding to a pre-existing cervical scar.

We found that VAM has several advantages for resection of ectopic parathyroid tissue. It has a short theatre time does not require a complex anaesthetic and is performed with the patient in classic supine position utilising often a previous cervical scar with good cosmetic results. It offers a short hospital stay; it is cost effective with minimal use of fancy and pricy consumables with a comfortable incision and no violation of the pleural space.

Additionally the use of digital Video imaging has increased the sensitivity of the mediastinoscopy and has added safety and confidence in performing a detailed mediastinal exploration with an additional great training value as well.

## Competing interests

The author(s) declare that they have no competing interests.
